# The Neuronal-Specific SGK1.1 (SGK1_v2) Kinase as a Transcriptional Modulator of *BAG4*, *Brox*, and *PPP1CB* Genes Expression

**DOI:** 10.3390/ijms16047462

**Published:** 2015-04-02

**Authors:** Rebeca González-Fernández, Julio Ávila, María F. Arteaga, Cecilia M. Canessa, Pablo Martín-Vasallo

**Affiliations:** 1 Laboratorio de Biología del Desarrollo, UD de Bioquímica y Biología Molecular and Centro de Investigaciones Biomédicas de Canarias (CIBICAN), Universidad de La Laguna, Av Astrofísico Sánchez s/n, La Laguna, Tenerife 38201, Spain; E-Mails: refernan@ull.es (R.G.-F.); javila@ull.es (J.A.); 2 Department of Cellular and Molecular Physiology, Yale University, 333 Cedar Street, New Haven, CT 06510, USA; E-Mails: marifrancis.arteaga@gmail.com (M.F.A.); cecilia.canessa@yale.edu (C.M.C.); 3 Department of Medicine A, Hematology, Hemostaseology, Oncology and Pneumology, University of Muenster, Muenster 48149, Germany; 4 Department of Basic Medical Sciences, Tsinghua University School of Medicine, Beijing 100084, China

**Keywords:** SGK1.1, transcription regulation, subtractive hybridization, transcriptional coactivator, Brox1

## Abstract

The Serum- and Glucocorticoid-induced Kinase 1, SGK1, exhibits a broad range of cellular functions that include regulation of the number of ion channels in plasma membrane and modulation of signaling pathways of cell survival. This diversity of functions is made possible by various regulatory processes acting upon the *SGK1* gene, giving rise to various isoforms: SGK1_v1–5, each with distinct properties and distinct aminotermini that serve to target proteins to different subcellular compartments. Among cellular effects of SGK1 expression is to indirectly modulate gene transcription by phosphorylating transcriptional factors of the FOXO family. Here we examined if SGK1.1 (SGK1_v2; NM_001143676), which associates primarily to the plasma membrane, is also able to regulate gene expression. Using a differential gene expression approach we identified six genes upregulated by SGK1.1 in HeLa cells. Further analysis of transcript and protein levels validated two genes: *BCL2-associated athanogene 4* (*BAG-4*) and *Brox*. The results indicate that SGK1.1 regulates gene transcription upon a different set of genes some of which participate in cell survival pathways (*BAG-4*) and others in intracellular vesicular traffic (*Brox*).

## 1. Introduction

The human and mouse genomes contain three independent genes coding for closely related protein families known as SGK1, SGK2, and SGK3 [1,2]. SGK1 participates in many different signaling pathways. The most studied is modulation of ion channels like the epithelial sodium channel, ENaC [3,4] and the voltage gated K^+^ channel Kv [5,6]. SGK1 either directly phosphorylates the channel proteins [7] or regulates their abundance in the plasma membrane through modulating the activity of the E3 ubiquitin ligase Nedd4-2 [8,9]. In addition, SGK1 also participates in various cell survival signaling pathways through the regulation of transcriptional factors like FKHRL1 [10,11], p53 [12,13], NF-κB [14,15] or β-catenin [16].

Participation of SGK1 in such a broad spectrum of cellular functions is made possible by a multilayer system of regulation that includes regulation of gene transcription by different promoters, differential splicing of transcripts that generates various isoforms (SGK1_v1–5), phosphorylation of the kinase domain that increases enzymatic activity, and specific subcellular localization of isoforms within cells [17,18,19,20,21,22]. SGK1.1 (SGK1_v2; NM_001143676) is a splice isoform conserved from rodent to humans and highly expressed in the nervous system [23,24]. These neuronal isoform differ from SGK1 in the amino terminus, which determines higher protein stability [23]. Despite a preferential localization of some SGK1 isoforms to specific subcellular compartments (SGK1 associates to endoplasmic reticulum membranes and SGK1.1 to the plasma membrane) all isoforms travel to the nucleus either constitutively or in response to external stimuli [25]. For instance, the aminoterminus of the SGK1.1 isoform contains a cluster of large hydrophobic and positively charged residues that together form a motif that binds PtdIns(4,5)P_2_ thereby sending SGK1.1 to the plasma membrane in resting cells [23]. Hydrolysis of PtdIns(4,5)P_2_ by phospholipase C triggers rapid translocation of SGK1.1 to the cytosol and nucleus raising the possibility that SGK1.1 may regulate transcription of genes involved in channel function or in cell survival [23].

The mutant SGK1.1S515D carries a negatively charged residue in the carboxyterminal hydrophobic motif where serine 515 is phosphorylated by PDK1 (3-phosphoinositide dependent protein kinase 1); this phosphorylation renders the kinase enzymatically active. Introduction of a negative residue, aspartic acid in position 515, mimics permanent phosphorylation thereby making the SGK1.1 protein constitutively active. In the mutant SGK1.1K220A lysine 220, located in the ATP binding site, has been replaced by alanine to eliminate enzymatic activity thus, this mutation is a non-active kinase [23,26].

We examined the differential gene expression induced in HeLa cells [27] by the constitutively active form SGK1.1_S515D_ *vs.* the inactive form SGK1.1_K220A_.

## 2. Results and Discussion

Transfection of SGK1.1 mutants SGK1.1_S515D_ and SGK1.1_K220A_ in HeLa cells was performed with similar efficiency of expression at the protein level.

### 2.1. Screening the Subtracted cDNA Library Identified Six Independent and Differentially Expressed Clones

A subtraction was performed in order to generate a library enriched in cDNAs specifically activated by SGK1.1. The hybridization procedure separates differentially expressed cDNAs and excludes those common to cells transfected with active SGK1.1_S515D_ and those transfected with non-active SGK1.1_K220A_ forms. Screening of 960 clones yielded 27 that were differentially expressed between the two conditions. To confirm that those selected clones were expressed in different amounts, PCR amplified DNA was subjected to dot blot as stated in “Materials and Methods” section. Expression differences were confirmed in all 27 clones. DNA sequencing of these clones identified six independent genes while the other clones contained segments of the cloning vector. The genes were: *BCL2-associated athanogene 4* (*BAG4*, NM_001204878.1) found in one clone; *Pongo abelii* BRO1 domain and CAAX motif containing (*Brox*, NM_144695.3) was found in three clones; radixin (*RDX*, NM_001260492.1) in one clone; lysocardiolipin acyltransferase (*LYCAT*, NM_001002257.1) in two clones; protein phosphatase 1, catalytic subunit, beta isozyme (*PPP1CB*, NM_002709.2) in one; and chromosome 6 open reading frame 62 (*C6ORF62*, XM_005249433.1) in one clone.

### 2.2. Anti-Brox Antibody (Brox-Ab) Specificity

**Figure 1:**
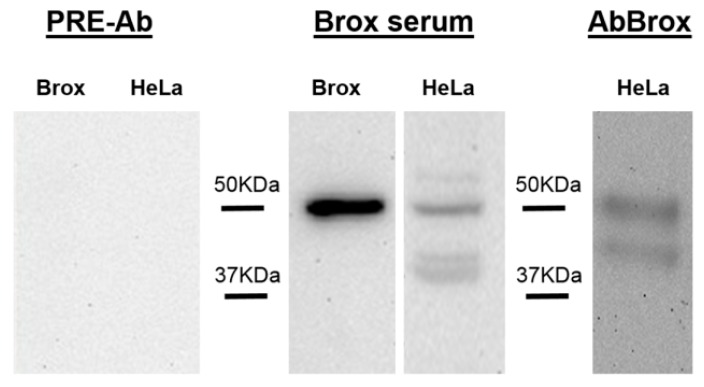
Western blot of HeLa cells lysates and purified recombinant Brox protein (Brox) probed with pre-immune serum (PRE-Ab), Brox serum and purified Brox specific antibodies (AbBrox).

In order to check the specificity of the antibody generated against recombinant Brox, the serum was used to probe in Western blots the *E. coli* recombinant protein and HeLa cells lysates. Additionally, pre-immune serum was also checked. Pre-immune serum did not recognize the recombinant protein used for the immunization or any protein in HeLa cells lysates (Figure 1). Brox anti-serum recognized the recombinant protein and identified four bands in HeLa cells, one of these with the expected molecular weight, 50 kDa. To further test the specific Brox band, we purified the serum as detailed in the Materials and Methods section. After purification, AbBrox antibody recognized two proteins identified as Brox-1 and Brox-2 of 50 and 40 kDa, respectively (Figure 1).

### 2.3. Validation of Genes Identified by Subtraction Screening

To verify reproducibility of genes identified by the subtraction method, we assessed their level of transcription by qRT-PCR in SGK1.1 transfected HeLa cells using specific primers for each of the identified genes. Twenty different batches of transfected cells (SGK1.1_S515D_ *vs.* SGK1.1_K220A_) were analyzed. In addition, as a control for transfection efficiency, SGK1.1 expression was measured in all twenty samples. Because there is not endogenous expression of SGK1.1 in HeLa cells, no expression was found in non-transfected cells (1.5 ± 1.3) compared to SGK1.1_S515D_ (152,235.4 ± 186,661.0) and SGK1.1_K220A_ (36,030.0 ± 32,508.8) transfected cells.

From the six identified genes all showed increased expression levels in cells transfected with active SGK1.1 except for *C6ORF62* whose increase was not statistically significant (Table 1). On the whole, these results suggest SGK1.1_S515D_ up regulates expression of the remaining five genes.

**Table 1:** Quantification of mRNA expression of identified genes by qRT-PCR in transfected HeLa cells. Mean ± standard deviation for *BAG-4*, *Brox*, *LYCAT*, *PPP1CB*, *RDX* and *C6ORF62* gene expression levels are expressed as ×10^5^ relative to β-actin expression in SGK1.1S515D (active) and SGK1.1K220A (non-active) transfected cells. *p* value column shows the bilateral significance for each mean comparison using *t* student test (statistically significant difference *p* < 0.05).

Gene	SGK1.1_S515D_	SGK1.1_K220A_	*p* Value
*BAG4*	96.5 ± 62.1	41.3 ± 27.3	0.001
*Brox*	178.8 ± 101.9	81.7 ± 61.6	0.001
*LYCAT*	319.9 ± 210.1	196.6 ± 117.4	0.029
*PPP1CB*	2492.9 ± 1625.4	1528.0 ± 832.2	0.023
*RDX*	1355.0 ± 961.6	829.7 ± 638.5	0.042
*C6ORF62*	4684.2 ± 2856.7	4341.8 ± 3026.0	0.715

**Figure 2:**
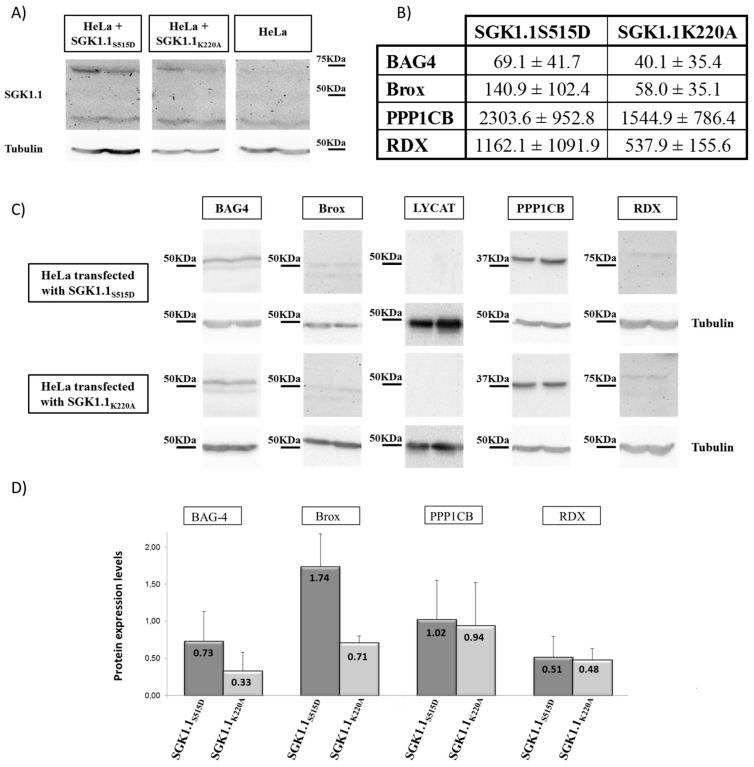
Western blots (**A**,**C**), quantification of mRNA relative expression (**B**) and bar chart of protein expression (**D**) of BAG-4, Brox, LYCAT, PPP1CB and RDX of same eight HeLa cultures of transfected cells used for (**D**) data. (**A**) Examples of SGK1.1 expression in transfected and no transfected HeLa cells and tubulin reference; (**B**) Mean ± standard deviation for *BAG-4*, *Brox*, *PPP1CB* and *RDX* gene expression as ×10^5^ relative to β-actin; (**C**) Examples of quantitated proteins and tubulin reference; (**D**) BAG-4, Brox, PPP1CB and RDX proteins expression levels relative to β-tubulin shown as mean ± standard deviation.

We next examined expression of the identified genes at the protein level by Western blot using commercially available antibodies and β-tubulin as reference for relative expression (Figure 2). Panel A shows an example of two Western blot lines from the eight Western blots performed for each protein in active and non-active transfected cells. Panel B shows mean and typical error of BAG4, Brox, PPP1CB and RDX relative protein expression in SGK1.1_S515D_ and SGK1.1_K220A_ transfected cells. Bars in chart (Figure 2, panel B) represent the mean of eight independent experiments.

No LYCAT expression at the protein level was observed in HeLa cells, thus no densitometry analysis was performed (Western blot in Figure 2). SGK1.1 expression served as a control for transfection efficiency of each group SGK1.1_S515D_ = 2.10 ± 1.15; SGK1.1_K220A_ = 1.78 ± 0.82; *vs.* non-transfected cells that gave a value of 0.23 ± 0.09 relative to β-tubulin expression.

Endogenous BAG4 and Brox proteins expressed at higher levels in the presence of SGK1.1_S515D_ than SGK1.1_K220A_ while no significant difference was observed with PPP1CB and RDX. These results are in agreement with the transcripts abundance in the same transfected cultures used for protein quantification (Figure 2, panel C), strengthening the validity of the subtraction screening. Exceptions are RDX and PPP1CB, whose transcript was increased in the eight samples (Figure 2, panel C), however, at the protein level the increment was much smaller, indicating that in the present model the final regulation of RDX and PPP1CB expression is much more complex than expected.

### 2.4. Immunolocalization in HeLa Cells

**Figure 3:**
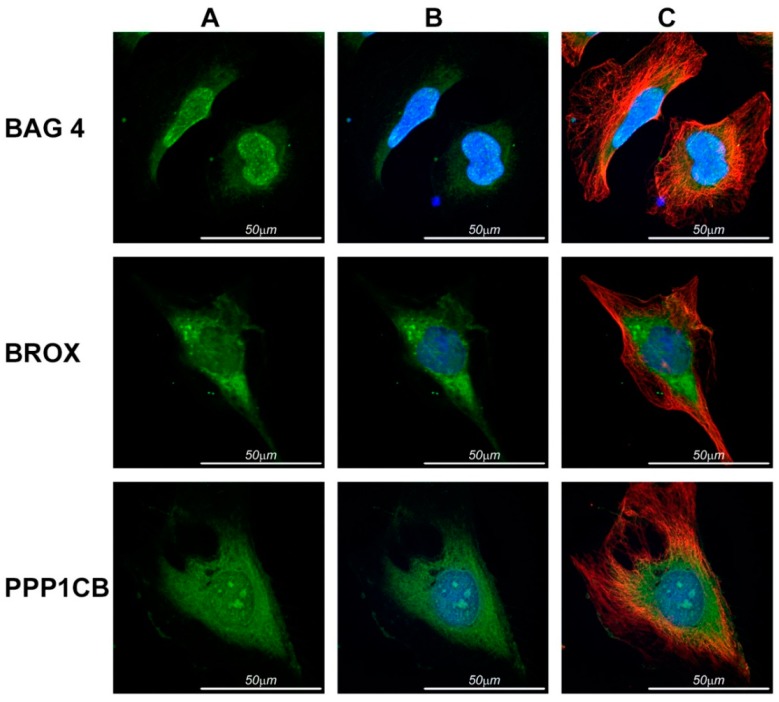
Subcellular localization of BAG4, Brox and PPP1CB in non-transfected HeLa cells. (**A**) Studied proteins using specifics antibodies are shown in green; (**B**) merge of studied proteins (green) and nucleus (blue) labeled with DAPI; (**C**) merge of studied proteins (green), nucleus (blue) and β tubulin (red).

Immunocytochemistry of endogenous BAG-4, Brox and PPP1CB proteins was performed on HeLa cells using antibodies specific for each protein and co-staining with DAPI and β-tubulin and confocal microscopy images taken (Figure 3).

Immunostaining of BAG4 was localized in nucleus and cytoplasm, mainly in the perinuclear area, extending as thin branches over the cytoplasm. Brox fluorescent signal was found around nuclei and also in the cytoplasm, where showed in a granular pattern suggestive of intracellular vesicles. PPP1CB signal was located throughout the cell in a punctum pattern.

**Figure 4:**
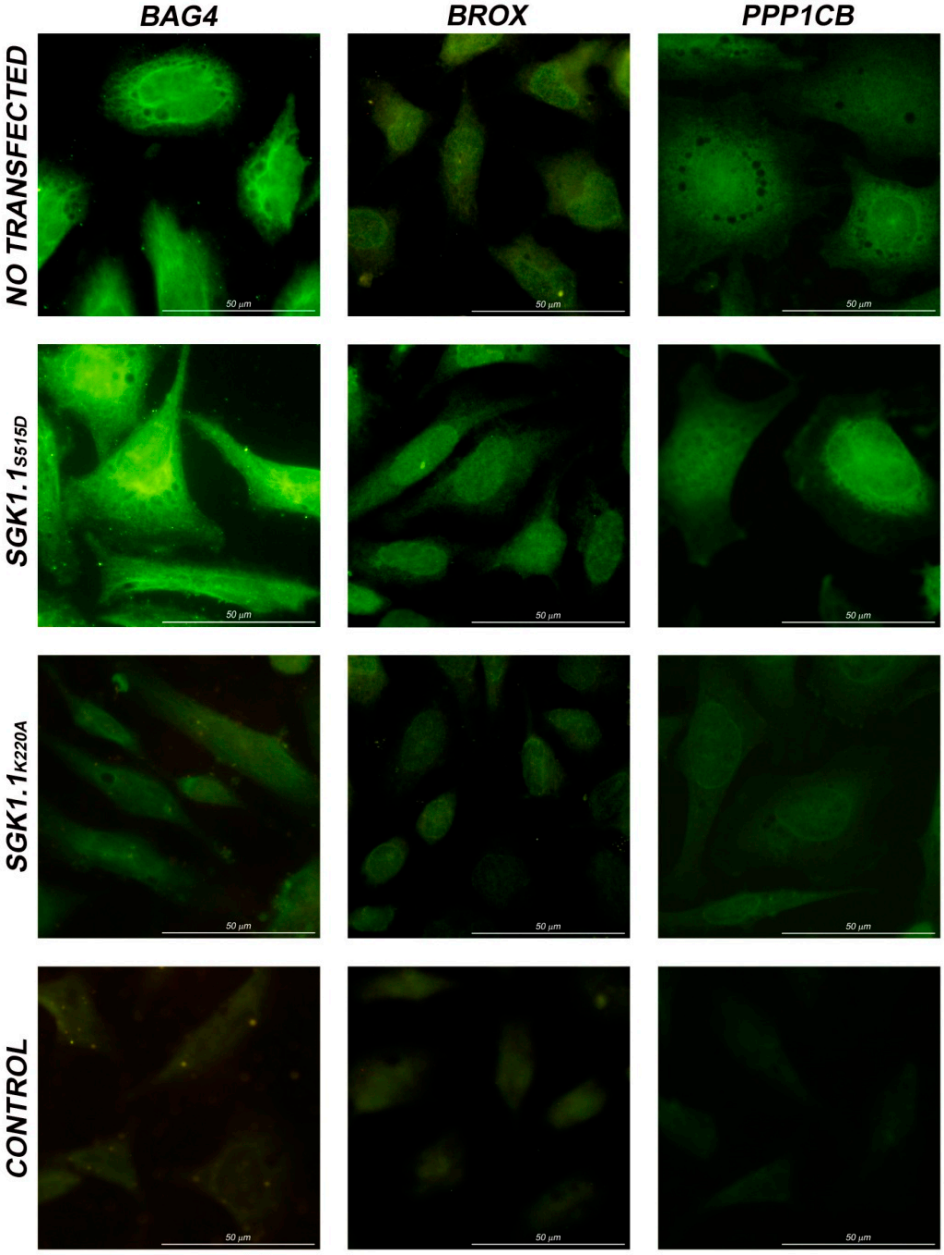
Immunolocalization of BAG-4, Brox and PPP1CB in non-transfected HeLa cells and SGK1.1S515D or SGK1.1K220A transfected HeLa cells. Negative control for secondary antibody at the same exposure time of the corresponding experiment.

Fluorescence intensity was higher in cells transfected with active SGK1.1 than in those transfected with the non-active form (Figure 4). All three proteins exhibited the same subcellular localization and pattern of expression in non-transfected and SGK1.1 transfected cells.

BAG4, Brox and PPP1CB expression in non-transfected cells is higher than in non-active SGK1.1 transfected cells. However, we must consider that SGK1.1_K127A_ is an enzymatically inactive mutant, but with the same structure and the same binding capacity that SGK1.1. Furthermore, as SGK1.1 modulates the expression of these genes, but not exclusively, the overexpression of the inactive form could interfere the action of other possible kinases also involved in transcriptional regulation by competing for the binding of common substrates.

To determine whether SGK1.1 also functions as a regulator of gene expression and to identify genes under transcriptional control by SGK1.1, we constructed and screened a differential subtraction library between cells expressing constitutively active and non-active SGK1.1 mutants. Six differentially expressed genes were identified in HeLa cells. We chose HeLa cells in order to avoid the effect of endogenous SKG1.1 background expression in the present study. We can then be sure that the differential changes are due to transfected SGK1.1. Further analysis validated five of those genes that were up-regulated at the transcription level: *BAG-4*, *Brox*, *LYCAT*, *PPP1CB* and *RDX*. At the protein level, BAG4 and Brox were validated by Western blot and immunohistochemistry analysis. In Western blot, differences in PPP1CB protein levels between active and non-active SGK1.1 transfected cells observed were small; however, these differences were clearly observed in immunocytochemistry experiments showing cell by cell contrasting staining levels. No change in subcellular distribution was observed. For Western blot we used the total cells obtained in the transfected culture in which not all the individual cells are transfected. No expression of LYCAT at the protein level was found using available antibodies. However, we confirmed that the antibody recognized LYCAT in liver and heart tissues samples (data not shown).

The identified and validated genes belong to families of proteins that have very different functions: cell survival by inhibiting signaling pathways that lead to apoptosis (BAG-4) [28,29], modulation of endosomal traffic (Brox) [30,31], and phosphatase activity (PPP1CB) [32]. The specific contributions of these proteins to the effects carried by SGK1.1, namely regulation of ion channel activity or cell survival, are not known yet but deserve further investigation. In particular, involvement of Brox in vesicular traffic is of great interest given the recognized importance of endocytosis and vesicular recycling in setting the number of ion channels at the plasma membrane.

Since SGK1.1 is a protein kinase and not a transcription factor, the most likely mechanism underlying the upregulation of gene expression is phosphorylation of a transcriptional factor or enhancer. Indeed, the isoform SGK1 phosphorylates and negatively regulates members of the FOXO family of transcription factors involved in promoting apoptosis [10,33]. No genes identified in the subtraction library have their transcription controlled by FOXO suggesting that SGK1.1 effects occur by targeting some other transcription factors different from FOXO. The difference might lie in the localization to the plasma membrane that exposes SGK1.1 to a different set of substrate molecules and a different mode of activation than those of the isoform SGK1.

In addition, it is possible that SGK1.1 targeted genes are cell specific. Consequently, a similar subtraction experiment conducted in other cell lines or tissues may lead to identifying additional genes. It would be worth conducting this experiment in cells that express endogenous ENaC, Kv1.3 or other ion channels regulated by SGK1.1 in order to identify novel regulatory genes.

SGK1 increases cell survival in development [14], T cell differentiation [34], and the proliferation of cancer cells; to a large extent—though not exclusively—by inhibiting apoptosis. The molecular pathways vary in different cell types and conditions. For instance, DNA damage induces SGK1 that by phosphorylating and inactivating the FOXO3a transcription factor increases survival [13], or by activating IкB kinase [15]. SGK1 phosphorylation of glycogen synthetase-3 leads to macrophage survival by stabilizing the antiapoptotic protein Mcl-1 [35]. Hence, SGK1.1 upregulation of BAG-4, a protein that binds to TNF-R1 and inhibits activation by TNFα, is in concordance with the overall antiapoptotic functions of SGK1.

SGK1 also regulates the ubiquitin ligase Nedd4 that promotes endocytosis of cell surface membrane proteins [36]. We found here that SGK1.1 upregulates Brox, a protein that binds to the endosomal sorting complex required for transport of ubiquitinated cargos into multivesicular bodies. Thus, SGK1 targets several steps in this pathway: ubiquitination of cargo at the plasma membrane and transport of endosomes containing ubiquitinated cargo for degradation in multivesicular bodies.

## 3. Experimental Section

### 3.1. Site Directed Mutagenesis

Mouse SGK1.1 cloned in pcDNA3.1/v5–His–TOPO (Invitrogen, Carlsbad, CA, USA) was used as the expression vector. Point mutations in SGK1.1 sequence were introduced using QuickChange Site-Directed Mutagenesis Kit (Stratagene, La Jolla, CA, USA) to generate the active mutant SGK1.1_S515D_ and the non-active mutant SGK1.1_K220A_ [23,26]. DNA sequencing confirmed presence of the introduced mutations.

### 3.2. Cell Culture and Transfection

HeLa cells were cultured in F12K (Kaighn’s modification of Ham’s F12) medium with 10% fetal calf serum at 37 °C, 5% CO_2_. Cells 90%–95% confluent were transfected with SGK1.1_S515D_, SGK1.1_K220A_, or with empty pcDNA 3.1 TOPO vector using Lipofectamin 2000 (Invitrogen, Carlsbad, CA, USA). The amount of DNA and lipofectamin used was half of that indicated in the manufacture instructions. By using half of the dose recommended we achieved equal transfection efficiency at increased cell viability. Twelve hours after transfection, culture medium was substituted for fresh medium with no fetal calf serum, and cells were incubated for four additional hours until collected in cold PBS (in mM): 137 NaCl, 2.7 KCl, 8 Na_2_HPO_4_, and 1.8 KH_2_PO_4_.

### 3.3. mRNA Extraction from Cells, Double Stranded cDNA Synthesis and PCR Amplification

HeLa cells were collected by centrifugation at 300× *g* for 5 min. mRNA from the pellet was extracted with Total Aurum RNA extraction kit (Bio–Rad, Mannheim, Germany), following the manufacturer’s instructions.

cDNA to be used in real-time quantitative RT-PCR (qRT-PCR) assay was synthesized using the iScript cDNA Synthesis kit (Bio–Rad, Mannheim, Germany). For the subtraction library we used the Super SMART PCR cDNA Synthesis kit (Clontech Laboratories, Mountain View, CA, USA), according to the manufacturer’s instructions. This kit uses PowerScript Reverse Transcriptase (Clontech), which allows synthesis of a high percentage of full-length cDNAs. Clean ds-cDNA was amplified by LD-PCR. Only full-length cDNA was amplified by using 5' PCR primer IIA, which hybridizes to the small sequence added during the cDNA synthesis process. Protocol was as follows: 95 °C for 60 s and X cycles of 95 °C for 15 s, 65 °C for 30 s and 68 °C for 6 min. X is the number of cycles previous to saturation, but enough for amplifying all cDNA of the sample in order to maintain the original ratio of gene expression.

### 3.4. ds-cDNA Subtraction and Library Construction

A subtraction library was prepared using the “Clontech PCR Select cDNA Subtraction Kit” (Clontech, Palo Alto, CA, USA) [37] following the manufacturer’s instructions using ds-cDNA from SGK1.1_S515D_ and SGK1.1_K220A_ transfected cells. The pool of differentially expressed Non-subtracted cDNAs from SGK1.1_S515D_ were cloned into pBluescript SK^+^ vector using T4 ligase (Promega, Madison, WI, USA) at 16 °C overnight and transformed into competent *E. coli* XL2-Blue cells by electroporation.

### 3.5. Dot-Blot Screening

Probes used were subtracted cDNA obtained from cells transfected with active and non-active SGK1.1 and digoxigenin-dUTP labelled (Roche Diagnostic, Indianapolis, IN, USA) using Klenow enzyme (Roche Diagnostic, Indianapolis, IN, USA).

Electroporated *E. coli* XL2-Blue cells were seeded in LB agar plates containing ampicillin (0.1 mg/mL), X-Gal (40 µg/mL) and IPTG (0.2 mM) and incubated at 37 °C overnight. Colonies containing insert were picked and placed into 96-well plates in liquid LB-ampicillin (0.1 mg/mL) medium and incubated at 37 °C overnight. A volume of 1 µL from each cultured well was placed by duplicate onto two different Roche (Roche Diagnostic, Indianapolis, IN, USA) nylon membranes and incubated at 37 °C overnight over LB-ampicillin (0.1 mg/mL) solid medium plates. Duplicate membranes were probed with both probes overnight at 68 °C. After washing and blocking, the membranes were incubated in Blocking solution containing Anti-Digoxigenin-AP (Roche Diagnostic, Indianapolis, IN, USA) for 30 min, washed twice and detected with CDP-Star (Roche Diagnostic, Indianapolis, IN, USA) as directed by manufacturer.

Positive clones were re-tested by double-dot-blotting of the PCR amplified product using T3 and T7 flanking sequences primers. Membranes were probed and detected as stated above.

### 3.6. DNA Sequencing and Sequence Identification

DNA from each clone was prepared with the Qiaprep kit (Qiagen, Hilden, Germany) following manufacturer’s instructions. DNA inserts were sequenced from both ends at La Laguna University DNA Sequencing Facility. Obtained DNA sequences were aligned to those in the GenBank, EMBL, DDBJ and PDB databases using the BLASTN and BLASTX algorithms [38].

### 3.7. Gene Expression Analysis by Quantitative RT-PCR (qRT-PCR)

All PCR reactions were carried out using a Bio–Rad CFX96 Real-Time PCR System (Bio–Rad, Hercules, CA, USA). Primers specific for each gene amplification were: BAG-4 (F = ACTGTCAGACTGAAGCACC; R = AACACTACGATTACCATCTCC), Brox (F = TTTGATCTCACCAAAAGACC; R = GATGTAGCACCCAGTGTCC), LYCAT (F = GCAACATGGCTCACCCTACC; R = ATTCCACAGGAACATCCAGTCC), PPP1CB (F = GGAGGATTGTCACCAGACC; R =AGACCATAGCAAATCACAGAGC), RDX (F = CGAGGAAGAACGTGTAACC; R = CCTGCTTTAACATTCTCAGC), C6ORF62 (F = TACAGCTCCATGCTCCTCG; R = GCTCATCAGATTCTTTCCACC) and SGK1.1 (F = CCCCAACTTGAAGTACACTGG; R = CATCCCCCTTTGGAAAGC). PCR was performed in a 10 μL final volume containing 2× SYBR Green Supermix (100 mM KCl, 40 mM Tris–HCL pH 8.4, 0.4 mM of each dNTP, iTaq DNA polymerase 50 units/mL, 6 mM MgCl_2_, SYBR Green I, 20 nM fluorescein, and stabilizers (Bio–Rad Laboratories, Hercules, CA, USA) and 10 μM of each primer.

Samples were analyzed in triplicate, and multiple water blanks were included in experiments. The thermal profile consisted on: 5 min denaturation at 95 °C, 40 cycles of PCR performed at 95 °C for 10 s, 59 °C for 20 s and 72 °C for 15 s, and melting curve program 65 to 95 °C with a heating rate of 0.1 °C/s and read every 0.5 °C. The housekeeping gene β-actin (F = CTTCCTTCCTGGGCATGG; R = GCCGCCAGACAGCACTGT) was amplified as a reference for mRNA quantification. Expression levels of each gene are presented as individual data points as 2^Δ*C*t^ [39]. All gene expression data are referred as ×10^5^ relative to β-actin expression.

### 3.8. Plasmid Construction and Preparation of Recombinant Brox Protein

Human Brox sequence was amplified by PCR from cDNA of HeLa cells using Phusion polymerase (Thermo Scientific, Waltham, MA, USA) following the manufacturer’s instructions. A 1275 pb BamHI/EcoR1I fragment representing the entire Brox coding region was amplified (Brox; F1 = CGGGATCCCGGAGAAAATGACCCATTGG, R1 = CGGAATTCCGCTGCTAGAGAAATTCTAAGTGC). Sequence fidelity was checked by sequencing after cloning into pRSET-c expression vector (Invitrogen, Carlsbad, CA, USA). The recombinant protein was expressed in *Escherichia coli* BL21(DE) cells by induction with 1 mM isopropyl-β-d-thiogalactopyranoside (IPTG) at 37 °C for 4 h. Purification of the recombinant protein was performed using HisTrap FF column (GE Healthcare, Little Chalfont, UK) following the manufacturer’s instructions.

### 3.9. Production and Purification of Polyclonal Antibodies against the Brox

Antiserum against human recombinant Brox was raised in a male New Zealand rabbit in the La Laguna University Animal Care Facility. Animal care and experimental procedures used for this work were approved by the Institutional Animal Care and Use Ethical Committee at the University of La Laguna, for founded projects FIS PI11/00114 (September 2011) and FIS PI12/00729 (September 2012). Before injecting the immunogen, pre-immune serum was collected and treated as described above for the Brox serum. The rabbit was injected subcutaneously with 0.5 mL (200 µg) of purified recombinant protein emulsified with an equal volume of Freund’s complete adjuvant. Two additional injections were given with a ten days interval, with 100 µg recombinant protein mixed with incomplete Freund’s adjuvant in same proportions. Ten days after the final injection, blood was collected and clotting allowed for one hour at 37 °C and overnight at 4 °C. The antiserum (Brox serum) was collected by centrifugation (7000× *g*, 5 min).

Purification of anti-Brox antibodies was performed using HisTrap FF column (GE Healthcare, Little Chalfont, UK) linked to Brox recombinant protein. After passing the serum through the column, specific antibodies retained were eluted with 100 mM glycine pH 2.5 (AbBrox). Neutral pH was restored by adding 1/10 of total volume of 1 M Tris buffer pH 8. Antibodies were further purified by ionic exchange chromatography in columns of protein A-Sepharosa from *S. aureus* (Sigma, St. Louis, MO, USA).

### 3.10. Protein Expression Analysis by Western Blot

Protein extract from homogenized cells were electrophoresed on a denaturing 12% polyacrylamide gel and transferred to Immobilon™-P membranes (Millipore, Bedford, MA, USA) by electroblotting. Membranes were blocked in PBS/5% BSA for 1 h. Protein detection was performed using available antibodies BAG-4 (SODD H300, 1:1000; Santa Cruz Biotechnology, Santa Cruz, CA, USA), Brox (AbBrox, 1:5000; P. Martín-Vasallo/J. Ávila), LYCAT (Anti-LCLAT1 antibody-*C*-terminal, 1:1000; Abcam, Cambridge, UK) PPP1CB (PPP1CB, 1:100,000; Abcam, Cambridge, UK), RDX (RDX 215R^+^, 1:1000; a gift from Dr Woodward, [40] ) and SGK1 (SGK ct, 1:5000; [3]. Secondary antibody was anti-rabbit Ig Horseradish peroxidase (GE Healthcare, Little Chalfont, UK) and secondary sheep anti-mouse (Sigma, St Louis, MO, USA). Detection was performed using ECL plus (GE Healthcare, Little Chalfont, UK) reagents according to the manufacturer’s instructions. Western blot quantification was measured using Quantity One Image software in a Molecular Imager, ChemiDoc XRS (Bio–Rad Laboratories, Hercules, CA, USA), as relative expression compared to β-tubulin (β Tubulin (37), 1:1000; Santa Cruz Biotechnology, Santa Cruz, CA, USA). ChemiDoc XRS software converts Western blot signals into digital data and allows us to measure the total signal intensity inside a defined area, subtracts local background, and quantifies the relative protein expression [41].

### 3.11. Immunofluorescence Microscopic Analyses

HeLa cells seeded on 15 mm coverslips pre-coated with poly l-lysine (Sigma, St. Louis, MO, USA), were fixed in methanol 100% for 6 min at −20 °C. After washing three times with PBS buffer, the cells was blocking with universal blocking buffer (1% BSA, 0.1% gelatine, 0.5% Triton X-100, 0.05% sodium azide, 0.01 M PBS pH 7.4). Then, cells were incubated for 1 h at room temperature with the primary antibody (BAG-4 1:500, AbBrox 1:500, PPP1CB 1:500, β-tubulin 1:150) diluted in blocking solution, washed three times with PBS buffer and incubated 1 h with Anti-rabbit IgG FITC conjugate (Sigma, St. Louis, MO, USA) and, in the co-staining experiments, with Anti-mouse IgG-Fc DyLight 650 (Abcam, Cambridge, UK) diluted in TBS-T (0.05 M Tris, 0.9% NaCl, 0.05% Tween 20, pH 8.4). Finally, the coverslips were mounted with glycerol: PBS 9:1 and analyzed under Olympus BX-50 fluorescence microscope (Olympus, Tokyo, Japan) or with ProLong Diamond Antifade Mountant with DAPI (Thermo Fisher Scientific Inc., Waltham, MA, USA) under Leica AF confocal microscope (Leica Microsystems CMS, Mannheim, Germany).

### 3.12. Statistical Analysis

Statistical analysis was performed using the SPSS 20 software (IBM, Madrid, Spain). Measure of statistical dispersion of mean difference was calculated as unpaired *t*-test analysis and *p* < 0.05 was considered statistically significant.

## 4. Conclusions

This work shows that the isoform SGK1.1 modulates transcription of genes that are distinct from those regulated by the more ubiquitous isoform SGK1: *BAG*, *Brox*, *RDX*, *LYCAT*, and *PPP1CB* genes. Most likely, the differential effect is mediated through phosphorylation of isoform-specific transcription factors and/or enhancers. The novel genes identified here participate in signaling pathways that promote cell survival and in modulating vesicular traffic, which has implications for shuttling ion channels to and from the plasma membrane.

This fact might play a role during development of neurodegenerative diseases and responses to noxious stimuli to the brain. However, this hypothesis needs to be tested further.
